# Supplementary material for: Validation of Fitbit Charge 4 for assessing sleep in Chinese patients with chronic insomnia: A comparison against polysomnography and actigraphy
Source: PLoS One. 2022 Oct 18;17(10):e0275287. doi: 10.1371/journal.pone.0275287 (PMC9578631; doi:10.1371/journal.pone.0275287)
Supplement: Attachment — Submitted filename: Response to Reviewers.doc [file pone.0275287.s001.doc]

Thank the reviewers for the kind suggestions. We revised the manuscript in accordance with the reviewers’ comments. We acknowledge the reviewer’s comments and suggestions very much, which are valuable in improving the quality of our manuscript.

Here below is our description on revision according to the reviewers’ comments.

**Reviewer #1: The paper presents a validation study of a consumer sleep tracker, Fitbit Charge 4against two other methods. One method is the reference (gold) standard polysomnography, and the other is an often used medical device, actigraphy. The validation was performed in 37 patients suffering from insomnia. This is an important research question because more and more patients come to sleep centers and show their Fitbit results to a sleep physician in order to ask for a polysomnography.**
Reply: Thanks a lot for the reviewer’s comment.

**In the abstract and in the text, there is an error: SOL is not sleep of latency but sleep onset latency.**
Reply: Thanks a lot for the reviewer’s comment. We consider that the suggestion is very essential. We have revised this mistake.

**Paragraph 2.3: For the sleep recording a few more technical details are missing. You say, that a computerized sleep recorder by Embla was used. However, there are many different versions marketed. Please specify exactly which type (name) of hardware and which software (Somnologica, Remlogic) including version number had been used here. If different sleep recorders had been used, please mention this.**
Reply: Thanks a lot for the reviewer’s comment. We consider that the suggestion is very essential. We had added the hardware and software information in the main document. Sleep stages were automatically scored in 30-epochs using Remlogic 3.4.1(Embla Systems, Kanata, Ontario, Canada), and visually reviewed by a trained technicians following criteria set by the AASM Scoring Manual Version 2.2 to ensure accuracy of the staging. We will add this in the main document.

# I am happy that you specified the version number for the Actiware software. The same is required for the other software involved.

Reply: Thanks a lot for the reviewer’s comment. We consider that the suggestion is very essential. We added the version number for the Fitbit Charge 4, SPSS software and MedCalc software in the main document.

**Paragraph 2.4: For the sleep scoring, you say a trained neurophysiologist scored the PSG records according to AASM. However, there are several versions of AASM with important differences. Please specify exactly which AASM version was used.**

Reply: Thanks a lot for the reviewer’s comment. We consider that the suggestion is very essential. The AASM Scoring Manual version 2.2 was used. We will add this in the main document.

**Paragraph 2.4: Regarding the visual scoring by the trained neurophysiologist, I like to know the inter- and intra-scorer reliability of the person. This is, because we know that there is always a limited accuracy in the visual scoring.**
Reply: Thanks a lot for the reviewer’s comment. We consider that the suggestion is very essential. A trained neurophysiologist, who was an International Registered Polysomnography Technologist(RPSGT), scored the PSG records using 30-second epochs according to the AASM Scoring Manual Version 2.2. Since there is only one Polysomnography Technologist in the sleep laboratory, we could not get the inter-rater reliability. After reanalyzed the REM percentage conducted by PSG between the baseline and five-month follow up of 10 patients The intra-scorer reliability is 94.8%.

**Because the subjects of your study suffered from insomnia, I would like to know their ISI (Insomnia severity index) score. Did you assess this important value?**
Reply: Thanks a lot for the reviewer’s comment. We consider that the suggestion is very important. All the participants were evaluated with insomnia severity index when they were diagnosed as insomnia. ISI scores were obtained according to medical records after getting permission from the hospital. We added the ISI scores in the Results 3.1.

**How many nights did you perform polysomnography? Usually the first night is used for adaption and the second night is used for evaluation. How long did the patients wear the Actiwatch? Usually this is for two weeks. How long did the patients wear the Fitbit. Often this is for months.**
Reply: Thanks a lot for the reviewer’s comment. We consider that the suggestion is very important. Considering that all the insomnia participants received PSG to get a diagnosis in the same sleep laboratory, only one night of PSG was conducted just as most of the similar researches. Each participant wore the Fitbit and Actiwatch on the same non-dominant hand at the same time that the PSG was being performed.

# How did you take care of synchronization of the data between the three systems? I would like to know how accurate was the synchronization of a specific epoch? Each epoch is 30 seconds and a synchronization error of perhaps 5 or 10 seconds could give different scoring results. Reply: Thanks a lot for the reviewer’s comment. We consider this is a good question. Firstly, we keep the time of the three devices highly consistent. Secondly, the times at which the lights were turned on and off for the PSG were chosen to be the beginning and ending points for the AWS and FBC. As each patient was monitored for at least 8 hours, which is 960 epochs. In order to assess potential biases in PSG, FBC and AWS data synchronization, we calculate EBE specificity by sliding the epoch alignment by up to 90s(1-3 epochs forwards and backwards) .

# Was the Fitbit and the Actiwatch mounted on the same arm or different arms? This might result in some differences? Reply: Thanks a lot for the reviewer’s comment. We consider this is a good question. Each participant wore the Fitbit and Actiwatch on the same non-dominant hand at the same time that the PSG was being performed, which was in Methods 2.3.

**The number of patients is relatively small. Why did you investigate 37 patients only? There are publications which require that validation studies for devices recording sleep should have at least 64 patients.**

Reply: Thanks a lot for the reviewer’s comment. We consider this is a good question. Only 37 participants met our included criteria from October 2021 and February 2022. A systematic review assessed sleep parameters and stages and 22 articles qualified for systematic review, which found that sample size varied substantially between investigations, from 7 to 63 (median 30) participants, with approximately 77% of them involving more than 20 individuals. The reference is listed below. Further research should expand the sample size.

HAGHAYEGH S., KHOSHNEVIS S., SMOLENSKY M. H., et al. Accuracy of Wristband Fitbit Models in Assessing Sleep: Systematic Review and Meta-Analysis[J]. J Med Internet Res. 2019;21(11):e16273.

**Presentation of the results is good. Especially the Bland-Altman plots show the limited value of the indirect devices. I do find the AWS against FBC plots most interesting. Table 3 is a good summary of comparisons.**
Reply: Thanks a lot for the reviewer’s comment. We consider this is a good question.

**The discussion is well to the different points. I would recommend to structure it more to the different comparisons and also to show limitations better.**

Reply: Thanks a lot for the reviewer’s comment. We consider this is a good question. We had revised this in the main document.

**Reviewer #2: This is a well-written paper, which addresses an important issue, the measurement of sleep with new technologies.
The authors should think about presenting Bland-Altman-Plots, sensitivity, specificity, and accuracy data just for the comparison of the Fitbit with PSG as well as for the AWS with PSG. The reason for omitting the comparison Fitbit with AWS is that none of these devices could be assumed to be the “reference” of sleep measurement.**

Reply: Thanks a lot for the reviewer’s comment. We consider that the suggestion is very important. AWS is research-grade actigraphy measuring sleep and wakefulness. we presented sensitivity, specificity, and accuracy data for the comparison of the Fitbit with AWS in table 4. We also demonstrate Bland-Altmann-Plots to evaluate Fitbit against AWS for sleep-wake detection in Figure 3.

**On the other hand, it would be interesting to have a 4x4 table with the total numbers (or percentage) of epochs assigned to stages light sleep, deep sleep, dream sleep, and Wake illustrating the agreement of Fitbit with PSG for correct classification of sleep structure.**

Reply: Thanks a lot for the reviewer’s comment. We consider that the suggestion is very important. Since every participants had a 4x4 tables with the total numbers (or percentage) of epochs. it is impossible to show all the 4x4 tables in the main document. We will upload all the original data in the Science Data Bank for instead.

**Of course, wearables like the new Fitbit device have a great potential in assessing sleep in big populations. Although the authors should also discuss some limitations of those devices:
As they are no medical devices and most of them do not tend to become one manufacturers typically do not specify sensors, the algorithms used for data processing, statistical analysis, and reporting. This limit the interpretability and validity of results. Examples are the facts that authors had to collect sleep stages manually from summary figures and that without reason 8.1% of Fitbit recordings were unavailable for analysis.**

**Another problem with consumer devices is that if one device was tested in a study upon publication of results it might be replaced on the market by a new one with different hardware as well as software already. An example the authors stated is the Fitbit device with five (!) previous versions. A successor - the Fitbit Charge5 - is on the market already …
In summary this make it impossible to perform large studies in different cohorts or even to perform replication studies.**

Reply: Thanks a lot for the reviewer’s comment. We consider that the suggestion is very important. We will include all those limitations in our study. Even though one consumer device was tested in a study upon publication of results it might be replaced on the market by a new one with different hardware as well as software already, more and more researches have proved that a new consumer device showed better sensitivity, specificity and accuracy. Although our research shows that the FBC cannot completely replace the PSG in the quantification of sleep variables and the classification of sleep stage in patients with chronic insomnia disorder, the user-friendly and low-cost wearables do show some comparable functions to the PSG.

**Results, 3.3: prevent redundancy of data presentation (in text as well as in table 4)
Table 2: I suggest to either put demographics into extra table or in text.**

Reply: Thanks a lot for the reviewer’s comment. We consider that the suggestion is very important. We removed duplicate data in the results 3.3. Also, we put demographics in the text and removed demographics in the Table 2.
